# The epidemiology, etiology, and future prophylactic options for cancers in Mainland China

**DOI:** 10.3389/fonc.2025.1579378

**Published:** 2025-05-28

**Authors:** Hongsen Chen, Jie Xu, Wenbin Liu, Xi Chen, Ping Li, Guangwen Cao

**Affiliations:** ^1^ Shanghai Key Laboratory of Medical Bioprotection, Second Military Medical University, Shanghai, China; ^2^ Department of Epidemiology, Second Military Medical University, Shanghai, China; ^3^ Key Laboratory of Biological Defense, Ministry of Education, Shanghai, China; ^4^ Center of International Scholar Exchange, Second Military Medical University, Shanghai, China

**Keywords:** China’s cancer burden, cancer epidemiology, prevention strategies, chronic inflammation, modifiable risk factors, systemic interventions

## Abstract

Mainland China accounts for 18.66% of the world’s population, 24.17% of global cancer new cases, and 26.44% cancer-related death worldwide in 2022. We aim to characterize the spatiotemporal distribution patterns of cancer burden, elucidate the main causes of high cancer burden, and propose evidence-based strategies for the prevention and control of major cancer types in Mainland China. We conducted a systematic search for relevant articles in PubMed and CNKI. We also analyzed the related data from two key databases: the 2022 dataset of the International Agency for Research on Cancer (IARC) and the records of China’s National Mortality Surveillance System (from 2004 - 2018). Lung cancer, primary liver cancer (PLC), gastric cancer, colorectal cancer (CRC), and esophageal cancer accounted for 67.50% of all cancer-related deaths. Age-standardized incidence rates (ASIR) and mortality rates (ASMR) of PLC, gastric cancer, and esophageal cancer showed downward trends, while their crude rates kept increasing. The ASMR of lung cancer kept decreasing in urban but increasing in rural populations. The burden of CRC kept increasing. Increase in cancer incidence could be attributed to the co-existence of the poverty-related risk factors like chronic infections and affluence-related ones like metabolic disorders. Primary prevention targeting to these modifiable risk factors is cost-effective. Aerobic exercise could decrease cancer occurrence and cancer-related death via decreasing systemic low-grade inflammation. The second and tertiary prophylactic options should be epidemiologically optimized. Targeting the major risk factors co-existed during economic transformation should be a cost-effective strategy to decrease cancer burden in transforming countries.

## 1 The incidence and mortality of cancers

Globally, there were close to 20 million new cases of cancer in 2020 (1). Among them, lung cancer was responsible for about 2.5 million newly diagnosed cases, accounting for 12.4% of all new cancer cases globally, followed by female breast cancer (11.6%), colorectal cancer (CRC) (9.6%), prostate cancer (7.3%), stomach cancer (4.9%), and primary liver cancer (PLC) (4.3%) (**Table 1**).

**Table 1:** New cases and death of top 10 cancers in the world.

Cancer site	Incidence			Mortality		
	New cases	% of all sites	Rank	Deaths	% of all sites	Rank
lung	2 480 675	12.4	1	1,817,172	18.7	1
Female breast	2,308,897	11.6	2	665,684	6.9	4
Colorectum	1,926,118	9.6	3	903,859	9.3	2
Prostate	1,466,680	7.3	4	396,792	4.1	8
Stomach	968,350	4.9	5	659,853	6.8	5
Liver	865,269	4.3	6	757,948	7.8	3
Thyroid	821,173	4.1	7	47,485	0.5	24
Cervix uteri	661,021	3.3	8	348,189	3.6	9
Bladder	613,791	3.1	9	220,349	2.3	13
Non-Hodgkin lymphoma	553,010	2.8	10	250,475	2.6	11

Source: GLOBOCAN 2022, https://www.gco.iarc.fr/en/projects#database, accessed on 3 February, 2025.

Mainland China was projected to have 4.825 million new cancer cases, which account for 24.17% of the global cases in 2022 (2). Lung cancer, CRC, thyroid cancer, PLC, and stomach cancer are the top five most common malignant tumors, accounting for 57.42% of all new malignant tumors. Current data anticipate an estimated 35 million new cancer cases globally by 2050, a projection that poses an increasingly severe economic and social burden. According to the national registration data, the number of new cancer cases has been increasing annually in Mainland China. In 1980, there were 1.17 million patients with malignant tumors in Mainland China, primarily consisting of stomach cancer, PLC, esophageal cancer, and cervical cancer. By 1985, this number had increased to 1.52 million, with rising incidence rates of lung cancer, CRC, and breast cancer. In 2015, there were approximately 4.29 million new cases of cancers, with lung cancer becoming the leading cause of incidence. In 2016, there were 4.06 million new cancer cases in Mainland China, with lung cancer, CRC, stomach cancer, PLC, and female breast cancer being the most common cancers, which accounted for 57% of all new cancer cases. Between 2000 and 2015, the ASIR of esophageal cancer, stomach cancer, and PLC significantly decreased, whereas the ASIR of CRC in the entire population, as well as ASIR of female lung cancer, female breast cancer, cervical cancer, and endometrial cancer, significantly increased (3).

Cancer has been the second leading cause of death since 2010 in Mainland China (4).In 2022, 9.737 million people died from malignant tumors. Among these deaths, 1.817 million were caused by lung cancer, representing 18.7% of all cancer-related deaths, followed by CRC (9.3%), PLC (7.8%), female breast cancer (6.9%), and stomach cancer (6.8%) (**Table 1**). The ASMR of esophageal cancer, stomach cancer, and PLC significantly decreased, while the ASMR of male CRC, male pancreatic cancer, and prostate cancer and ASMR of female breast cancer, cervical cancer, and female thyroid cancer significantly increased (3). In 2016, there were 2.414 million cancer-related deaths, with lung cancer, PLC, stomach cancer, CRC, and esophageal cancer being the most common causes of cancer death, which accounted for 69.30% of all cancer deaths. Although China’s population accounts for 18.66% of the global population, there were 2.574 million cancer-related deaths in 2022, accounting for 26.44% of the global total (2).With the number of cancer related deaths increasing, the cost of cancer diagnosis and treatment in Mainland China reached 1.2 trillion yuan, with a per capita cost of 220,000 to 800,000 RMB (equal to 31,429 to 114,286 USD) in 2022. This high cost has caused a substantial economic burden on patients’ families and has become one of the main reasons for returning to poverty due to illness, exerting significant pressure on the national medical insurance system.

## 2 Main risk factors for the increasing cancer burden in Mainland China

**Figure 1:**
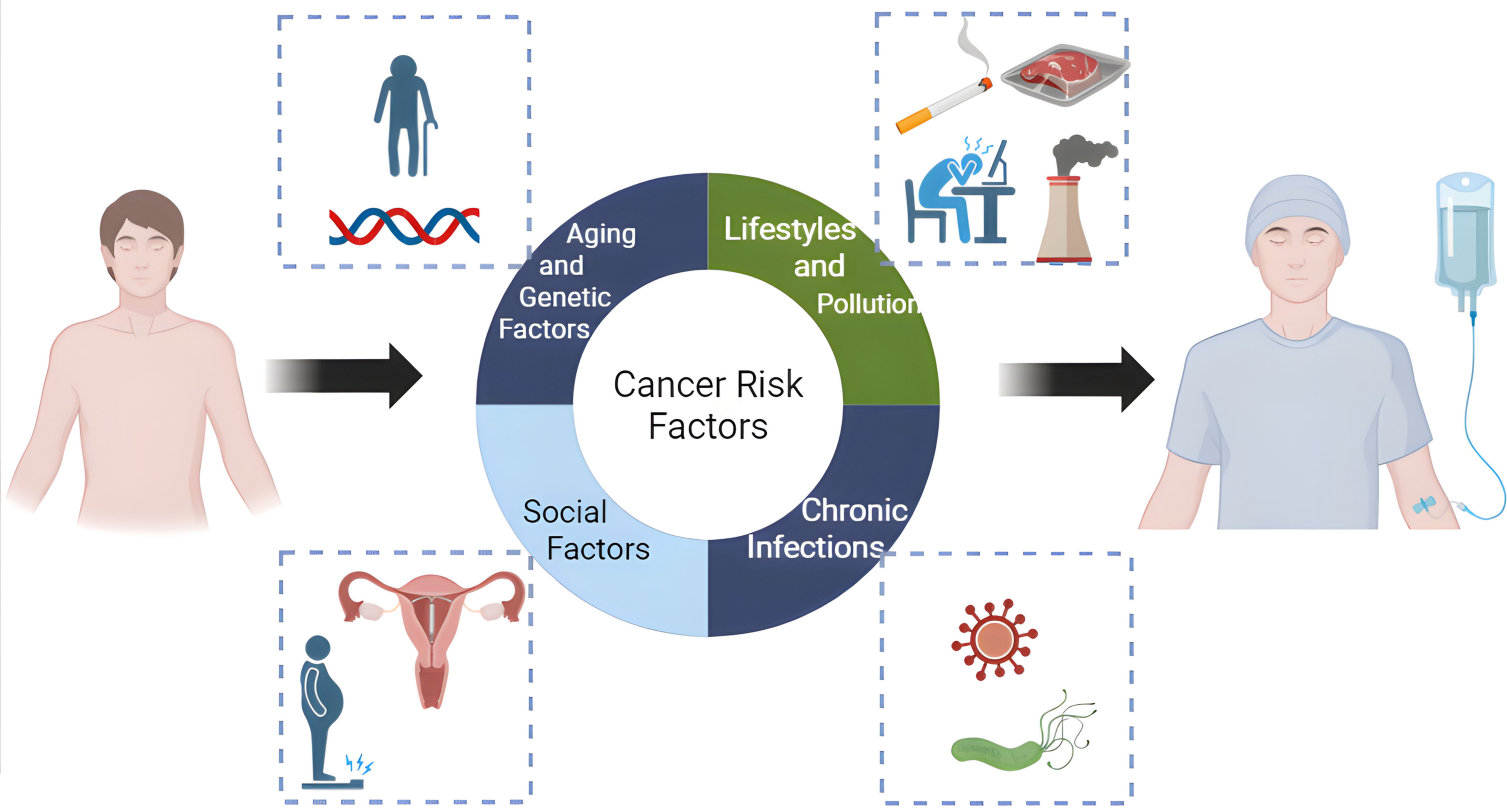
Main risk factors for the increasing cancer burden in China. (Figure was created with https://www.biorender.com, accessed on 5 Febrary, 2025).

During the socioeconomic transformation in Mainland China, improved socioeconomic situation-related cancer risk factors, including metabolic syndrome, physical inactivity, and alcohol consumption, keep increasing, while the poverty-related cancer risk factors, such as chronic infection and pollution, exist. In recent years, the increase in cancer incidence in Mainland China can be attributed to four major factors (**Figure 1**). The first is population aging and genetic susceptibility; the second is lifestyle and environmental exposures; the third lies between genetics and environment, namely chronic low-grade systemic inflammation; the fourth is social factors such as overdiagnosis, malnutrition and the application of contraception. Among them, the second to fourth causes can be attributed to modifiable risk factors which are the points for public health intervention, and serve as the main entry points for primary prophylaxis of cancers.

### 2.1 Aging and genetic factors

Aging represents the most critical risk factor for malignant diseases. The lifetime probability of being diagnosed with cancer increases progressively with increased age, reaching its peak between 65 and 84 years old in both male and female populations (5).With the progression of the society and the improvement in medical conditions, the aging burden in Mainland China has gradually increased (6), concomitantly driving corresponding changes in cancer mortality trends. An analysis of the leading causes of death in the Chinese population from 2004 to 2018 revealed significant increases in crude mortality rates for both cardiovascular and cerebrovascular diseases and malignant tumors, with these rises primarily attributed to population aging (7). Additionally, since 2000, whereas ASIR and ASMR for upper gastrointestinal tract tumors—including PLC, gastric cancer, and esophageal cancer—have demonstrated downward trends, ASIR and ASMR for lower gastrointestinal tract malignancies such as CRC and pancreatic cancer have exhibited annual increases (2, 7).

Human leukocyte antigen (HLA) - II genes influence the chronic infection and immune selection of hepatitis B virus (HBV) variants through immune mechanisms (8, 9). HLA-DRB1, HLA-DQA1, HLA-DQB1, and HLA-DPB1 loci are the most polymorphic and functionally significant in the HLA-II region. The most polymorphic HLA-DRB1 alleles can influence the susceptibility, resistance and prognosis of non-small cell lung cancer (NSCLC) (10, 11). It was found that the genetic susceptibility loci between HLA-DQB1 and HLA-DQA2 is significantly associated with increased risk of hepatocellular carcinoma through genome wide association study (12). HLA-DPB1 can facilitate Epstein-Barr virus (EBV) infection of B lymphocytes and jointly contributes to the development of Hodgkin lymphoma with EB virus (13, 14). HLA-DRB1 is associated with cervical cancer in Asian, Caucasian, Hispanic or Latin American and black sub-Saharan Africa populations (15).The genetic polymorphism susceptibility of HLA-II genes remains the primary genetic susceptibility in non-infectious malignant tumors such as lung cancer and pancreatic cancer, thereby promoting the maintenance of chronic inflammation and facilitating the evolutionary development of cancer (16).

### 2.2 Lifestyles

With the socioeconomic development and evolution of industrial division of labor, significant transformations have occurred in human lifestyles, including physical inactivity, smoking, excessive alcohol consumption, unhealthy diets, and increased psychological stress. While smoking and unhealthy diets are associated with direct carcinogenic effects, such as polycyclic aromatic hydrocarbons in tobacco smoke (which induce squamous cell lung cancer) and N-nitrosamines (which induce adenocarcinoma of the lung) (17), other lifestyle factors primarily induce low-grade chronic systemic inflammation in most cases. This inflammatory state promotes the progression and development of cancer (16). Lifestyle factors contributing to significant increases in cancer incidence and mortality represent the most critical modifiable risk factors and serve as a key entry point for controlling the progression of malignant tumors.

#### 2.2.1 Insufficient physical activity

The World Health Organization (WHO) defines physical activity as any bodily movement produced by skeletal muscles that requires energy expenditure (18). Physical inactivity represents the non-achievement of physical activity guidelines, and is one of the major public health issues today. Globally, nearly a third of adults, 1.8 billion adults, were insufficiently active physically (19), and 81% of adolescents (aged 11–17 years) were physically inactive (20).

In Mainland China, the elevated prevalence of obesity among Chinese adolescents has been attributed to the priority of academic performance over physical activity. Furthermore, the expanding service sector (tertiary industry) and increasing of “screen time” have transformed occupational patterns, with sedentary lifestyles becoming predominant among working adults. The standardized prevalence of physical inactivity has increased from 22.12% in 2013 to 28.79% in 2019 in Chinse adults (21). In Chinese adolescents, it was reported that the proportion of unhealthy lifestyle with respect to physical inactivity can reach 72.2% (22). WHO summarized global guidelines and evidence reviews exploring dose-response relationship or association between physical inactivity and health-related outcomes, and found that insufficient physical activity in individuals under 65 years of age was critical for adverse health outcomes including mental health disorders, adiposity, cognitive dysfunction, increased cancer incidence, type 2 diabetes, and cardiovascular disease (CVD) (23).

#### 2.2.2 Smoking

Tobacco products and burned smoke contain a complex mixture of more than 9500 chemical constituents, many of which have been identified as toxic or detrimental to human health. Notably, IARC has systematically classified 83 specific agents as carcinogens, including 37 in unburned tobacco and 80 in tobacco mainstream smoke (24).For example, the class 1 carcinogens classified by IARC are benzo[a]pyrene, which is causally linked to lung cancer (25), and 4-aminobiphenyl, which is a well-known bladder carcinogen (26). Smoking also has a definite effect on cancer risk of larynx, esophagus, pancreas, and kidney (27, 28). A meta-analysis of 29 mendelian randomization (MR) studies and 123 *de novo* MR analyzes showed that people with genetic liability to smoking (smoking initiation or lifetime smoking) were associated with increased risk of circulatory system diseases, several digestive system diseases, cancers of the lung, head and neck, esophagus, pancreas, bladder, kidney, cervix, and ovaries, and myeloid leukemia (29). Our cohort studies have confirmed that smoking is an independent risk factor for cancer incidence, particularly among individuals aged 65 and above (30). Significantly, prenatal and childhood/adolescent exposure to tobacco smoke, combined with genetic susceptibility, may significantly elevate the risks of lung cancer incidence and mortality in later adulthood (31).

Although e-cigarettes are promoted as harm-reducing alternatives to tobacco products, recent studies have revealed that e-cigarette use can have epigenetic effects of DNA methylation on oral, cervical, or blood cells and tissues, suggesting its role in cancer development. Therefore, a cautious attitude towards the use of e-cigarettes should still be maintained (32).

#### 2.2.3 Alcohol consumption

Several institutes including IARC have ascribed alcohol consumption as the highest level of causal evidence leading to the development of cancer (33, 34). On a global scale, it is estimated that in 2020, around 741,300 cancer-related death could be attributed to alcohol consumption. Among them, males made up 568,700 of the total alcohol-related cancer cases. The cancers of the esophagus, liver, and stomach were responsible for the largest of alcohol-attributable cases (35). Alcohol’s role as one of the leading causes of PLC can be manifested in alcoholic liver disease. Long-term alcohol use leads to continuous damage to liver cells, resulting in elevated levels of transaminases (36). In an attempt to repair this damage, the liver gradually develops alcoholic cirrhosis. Alcoholic liver disease contributes more to liver cancer than do other metabolic liver diseases (37). There are two primary biological mechanisms behind this: DNA damage and chronic inflammation caused by alcohol metabolites (38). First, two key enzymes are involved in alcohol metabolism: alcohol dehydrogenase 1B (ADH1B) and aldehyde dehydrogenase 2 (ALDH2). ADH1B converts ethanol into acetaldehyde, a potent carcinogen. Genetic variants in ADH1B (e.g., the ADH1B*2 allele) accelerate this conversion, leading to elevated acetaldehyde levels even at low alcohol doses (39). ALDH2 normally detoxifies acetaldehyde into harmless acetate. However, almost half of East Asians carry the loss-of-function ALDH2 rs671 variant (ALDH2*2 allele), which reduces acetaldehyde metabolism, causing dangerous accumulation of acetaldehyde in tissues (40).Excessive acetaldehyde production leads to DNA damage and significantly increases the risk of various malignant tumors, including liver cancer, esophageal cancer, and lung adenocarcinoma (41–43). Individuals with such genetic predispositions tend to experience vasodilation after drinking and can be easily identified; they are considered unsuitable for alcohol consumption (44). Adults should avoid liver damage from intoxication, mucosal damage in the upper digestive tract (such as the esophagus) from regular alcohol consumption, and chronic inflammation, such as persistent elevation of transaminases. Chronic liver inflammation indicates liver necrosis and proliferation. To limit this inflammation, the body encapsulates necrotic liver tissue with fibrosis, leading to alcoholic cirrhosis and ultimately resulting in inflammation-cancer transformation.

#### 2.2.4 Dietary factors

IARC Monographs Working Group has classified red meat and processed meat as significant carcinogenic risk factors, based on extensive studies of epidemiological evidences (45). A more recent systematic review and meta-analysis of prospective studies showed that high red meat intake was positively associated with risk of breast cancer, endometrial cancer, CRC, colon cancer, rectal cancer, lung cancer, and hepatocellular carcinoma and that high processed meat intake was positively associated with risk of breast, colorectal, colon, rectal, and lung cancers (46). Carcinogens including N-nitroso compounds and heterocyclic aromatic amines (HCAs) in red meat or processed food can be generated through pickling or high temperature cooking. These carcinogens can induce DNA damage, exacerbate chromosomal aberrations, activate oncogenic signaling pathways, and promote carcinogenesis (47).Polycyclic aromatic hydrocarbons (PAHs), tobacco specific nitrosamines, or aromatic amines generated from processed food materials via smoking, frying, barbecuing, improper fermentation, excessive heating, and spoilage are strongly associated with patient outcomes, tumor progression, the tumor immune microenvironment, programmed death ligand-1 (PD-L1) expression, and DNA damage in lung cancer (48). Additionally, red meat and processed meat may interact directly or indirectly on the epigenome, accelerating aging-related epigenetic alterations in cancer-associated genes and thereby influencing carcinogenesis (49).

Moreover, diets high in sugar, fat, and salt can easily lead to obesity, hypertension, and abnormal glucose metabolism. Unhealthy diet can lead to intestinal microbiota dysbiosis, which in turn increases the permeability of the intestinal barrier and induces liver inflammation, excessive production of reactive oxygen species (ROS), and synthesis of pro-inflammatory cytokines. Meanwhile, pro-inflammatory cytokines can activate monocytes and macrophages to produce ROS, thereby exacerbating the systemic inflammatory response and creating a microenvironment conducive to tumor growth in hepatocellular carcinoma (50). Consumption of scalding food can easily cause thermal injuries to the mucosal cells of the esophagus, causing repeated local inflammation, necrosis, and cell proliferation, and finally increases the risk of esophageal squamous cell cancer and stomach cancer (51).

#### 2.2.5 Anxiety and depression

From 1990 to 2021, the number of depressive disorder cases (from 34.4 to 53.1 million) and anxiety disorders (from 40.5 to 53.1 million) increased by approximately 54% and 31.2% respectively in Mainland China (52). Multiple epidemiological studies have established an association link between depression and anxiety with cancer incidence or mortality. A meta-analysis indicated that depression and anxiety are associated with a higher incidence risk of lung, oral, prostate, and skin cancers. Moreover, these mental problems are linked to an increased cancer-specific mortality risk for lung, bladder, breast, colorectal, hematopoietic system, kidney, and prostate cancers (53).Another cohort study showed that depression was associated with higher incidence risks of lung and CRC, but not breast or prostate cancer, and this association is independent of gender and age (54). Depression and anxiety often emerge in young adults and were associated with other potentially modifiable risk factors for cancers (55).

Meta-analyses, animal and clinical studies have demonstrated that depression can lead to elevated plasma inflammatory markers, such as interleukin-1 (IL-1), interleukin-2 (IL-2), interleukin-6 (IL-6), and tumor necrosis factor-alpha (TNF-α) (56–58). In individuals with major depressive disorder (MDD), the persistent maintenance of chronic inflammation and immune dysregulation may contribute to an increased risk of cancer development (52, 59, 60).

### 2.3 Environmental exposures

With the rapid economic development and industrial expansion in Mainland China since reform and opening up, environmental exposure to pollution has emerged as a critical challenge. Fossil fuel combustion releases substantial airborne pollutants, intensifying air quality deterioration. Concurrently, agricultural intensification has led to widespread soil contamination through excessive use of chemical fertilizers and pesticides. A national survey in Mainland China found that 19% of agricultural soils exceeded soil quality standards, with arsenic (As), cadmium (Cd), Cu, and nickel (Ni) accounting for the majority of exceedances (61). These environmental pollutions have led to a rise in cancer incidence in the 1990s. In the mid-1980s, to reduce urban pollution, some polluting enterprises were relocated from urban areas to suburban areas or rural regions. Our study on the changing trends of cancer mortality in Mainland China found that after accounting for the aging population, the ASMR of lung cancer, which is closely related to industrial pollution, gradually decreased in urban areas but increased in rural areas during 2004–2018 (7). This trend is more pronounced in the eastern regions of Mainland China, where industrial layouts are more concentrated. In the northern regions and rural southern areas, the use of solid fuels has significantly promoted the occurrence of lung cancer among non-smoking females (62). The main carcinogenic causes are persistent lung inflammation and dual stimulation by carcinogens such as benzo [a]pyrene (63).

A systematic review and meta-analysis illustrated a significantly inverse association between refrigerator use and the risk of gastric cancer in Asian countries. This protective effect may be attributed to improved food preservation methods that inhibit microbial growth and reduce nitrate-to-nitrite conversion in stored foods (64). This explains significant decrease in the incidence and mortality of gastric cancer in 1980s occurred when refrigerator widely came into Chinese families (65). Aflatoxin B1 (AFB1), an *Aspergillus flavus* metabolite, is an important food contaminant which often appears in humid areas. Food contamination with AFB1 is a major cause of HCC. Once metabolized by cytochrome P450, AFB1 produces aflatoxin B1-8,9-epoxide (AFB1-exo-8,9-epoxide), which is an extremely toxic substance that promotes the formation of DNA adducts and induces genomic mutations (66). In inland and southern regions of Mainland China, grains like peanuts and corn are prone to AFB1 contamination (67). In individuals infected with HBV, the carcinogenic effect of aflatoxin B1 is even more pronounced (68).

### 2.4 Chronic infections

The formation of chronic microbial infections is primarily due to that the immune system fails to clear invading pathogens while continuously attempting to eliminate them, leading to chronic inflammation, necrosis, and proliferation.

#### 2.4.1 HBV and HCV

In Mainland China, the chronic infection rate of HBV in adults is relatively high (7.18% in 2006). The number of individuals living with chronic HBV infection in Mainland China is about 75 million (69). Genotype C HBV is an independent risk factor for the chronicization of acute hepatitis B. Patients with chronic HBV infection mainly acquire the infection in infants and young children when their immune systems are not fully developed, with mother-to-child transmission being the most significant route. A higher risk of mother-to-child transmission is associated with HBeAg-positive mothers, younger maternal age, and high maternal HBV viral load. Antiviral treatment initiated after the 28^th^ week of pregnancy can significantly reduce the rate of mother-to-child transmission (70, 71). Chronic HBV infection is the leading cause of PLC globally. PLC mainly includes three histological types: HCC, intrahepatic cholangiocarcinoma (ICC), and mixed types, accounting for 93.0%, 4.3%, and 1.6%, respectively, in China. In China, the positivity rates of HBV and HCV in HCC cases are 84.4%and 3.2%, respectively. Compared with HBV-negative HCC, HBV-positive HCC occurs 10 years earlier and has a worse prognosis (72). To limit inflammation caused by HBV or hepatitis C virus (HCV), the human body encapsulates the inflamed tissue with fibrosis, leading to liver cirrhosis. Some pathogens, such as HBV, can evolve to acquire potential oncogenic properties (73).

HBV-induced carcinogenesis mainly relies on three factors: viral evolution, viral replication, and viral integration. HBV can evolve to acquire potential oncogenic properties (73). Viral evolution refers to the process that, after causing chronic infection, pro-inflammatory factors activate apolipoprotein B mRNA-editing catalytic polypeptide-like 3 (APOBEC3s) to promote viral mutations. The immune system’s selection of these mutated viruses significantly enhances their oncogenic potential (74, 75). The more active the viruses replicate, the greater the liver cells are damaged and inflamed. HBV infection not only promotes the occurrence and mortality of liver cancer but also increases the risk of hematological malignancies (76). HCV-induced carcinogenesis mainly occurs in Western countries and Japan. Since there is a lack of strong evidence for direct oncogenicity of HCV, cancer development is primarily through the induction of liver cirrhosis. Currently, antiviral drugs targeting HCV can achieve a cure, whereas HBV treatments can only suppress viral replication (77).

#### 2.4.2 Human papillomavirus

Persistent infections of high-risk HPV genotypes 16 and 18 are apt to catch cervical cancer (78). In southern China’s Guangzhou, HPV prevalence and genotype distribution were analyzed in 198,111 women undergoing cervical screening during 2015–2021. Overall, HPV positivity was 21.66% (42,911/198,111), with significant annual increases. Predominant genotypes included HPV 52, 16, 58. HPV positivity correlated with cervical intraepithelial neoplasia progression, with HPV 16 being the leading carcinogenic type, followed by HPV 52 and 18. Infections showed strong age-specific patterns, and 26.5% of positive cases involved multiple HPV types (79). Another retrospective analysis of genotyping results of 27 HPV types from treated patients between 2016 and 2021 revealed that among patients with cervical intraepithelial neoplasia grade II or higher (CINII+), 90.48% were HPV-positive, with HPV 33 and HPV 16 being the predominant types (80).

#### 2.4.3 EBV

EBV is transmitted primarily through saliva. Although the initial infection is often asymptomatic or causes mild, self-limiting illnesses, persistently infecting 90% of the global population (81). Epidemiological studies have illustrated the pathogenesis role of EBV with a series cancers, including nasopharyngeal carcinoma (82), gastric cancer (83), T-cell lymphoma (84), Hodgkin’s lymphoma (85), diffuse large B-cell lymphoma (86), and Burkitt’s lymphoma (87). The highest incidence of nasopharyngeal carcinoma is found in the Cantonese-speaking populations in Mainland China. This regional disparity is likely to be the result of a combined effect of the EBV subtypes involved, environmental factors, and genetic factors (88). It is currently believed that smoking activates EBV, leading to the occurrence and poor prognosis of nasopharyngeal carcinoma (89). Genetic susceptibility analysis has found that the HLA-II antigen HLA-DRB1*09:01 is significantly associated with the seropositivity rate of Zta-IgA, a marker of EBV activation (90), suggesting that inflammatory immune factors play an important role in activating EBV.

#### 2.4.4 Helicobacter pylori

*H.pylori* has been confirmed by epidemiological studies as the primary risk factor for gastric cancer (91). *H.pylori* triggers several histopathological changes in the gastric epithelium and maintains a sustained cascade of cytokines which in turn cause immune cell infiltration that generates oxidative radicals with the potential to damage host DNA (92). The cumulative DNA damage and epigenetic dysregulation drive histological progression along the “Correa cascade”, manifesting via chronic atrophic gastritis, intestinal metaplasia, and ultimately adenocarcinoma (93, 94). Eradication of *H.pylori* significantly decreases the incidence of gastric cancer only in populations with high genetic susceptibility (95). These suggest that gastric cancer is a typical inflammation-related tumor, and controlling inflammation can limit and reduce the disease burden of gastric cancer.

#### 2.4.5 Clonorchis sinensis

*Clonorchis sinensis*, *Opisthorchis viverrini*, and *Opisthorchis felineus* are the main species of liver flukes, and are widely distributed in Mainland China, Southeast Asia, and Eastern Europe, respectively. Mainland China has the highest number of *Clonorchiasis* infections globally, with approximately 11 million cases (96). The disease is mainly concentrated in the Pearl River Delta of Mainland China, including Guangxi, Guangdong (97), and the Songhua River basin in Heilongjiang and Jilin (98), primarily due to the consumption of raw and undercooked water or fish. The entire life cycle of the liver fluke involves multiple stages, including water, freshwater snails, freshwater fish/shrimp, and the definitive hosts (e.g., humans, cats, and dogs) (99). After entering the human body, the adult flukes mainly parasitize the intrahepatic bile ducts. The mechanical irritation caused by their feeding activities and the secretions/excretions induce chronic inflammation and periductal fibrosis of the bile ducts, leading to cholangiocarcinoma and liver cancer (100). In 2009, the IARC classified *Clonorchis sinensis* as a Group 1 carcinogen. Along with the HBV infection, liver flukes not only significantly increase the incidence but also promote postoperative recurrence and metastasis of liver cancer (101). Drug therapy is the main method for controlling liver fluke infections. Praziquantel is the only effective drug recommended by the WHO for the treatment of *Clonorchiasis* and is used in the main endemic countries (102). Avoiding the consumption of raw freshwater fish and shrimp and keeping stray cats and dogs in uncontaminated areas are the primary public health measures for controlling the carcinogenicity of *Clonorchis sinensis*.

### 2.5 Social factors

Prenatal famine exposure may induce compensatory overconsumption behaviors through developmental programming of psychological adaptation. This behavioral phenotype promotes excessive caloric intake, thereby exacerbating insulin resistance and systemic inflammation, which collectively contribute to the development of obesity, type 2 diabetes, and metabolic syndrome. These metabolic disorders are clinically associated with elevated risks of cancer incidence and mortality through multiple pathological pathways (103–105). Our previous research found that individuals born during the “Great Famine” of 1958–1962 had increased mortality rates from the four major chronic diseases, with the most significant increase in cancer mortality (106).

Nationwide cohort studies have confirmed that pregnancy can reduce endometrial cancer risk to around 40% independent of duration of pregnancy, age at pregnancy, or spontaneous abortions (107). This suggests that progesterone has a protective effect against endometrial cancer. The ASMR of endometrial cancer among Chinese women reached its peak in the 1990s and has been progressively declining over the past 20 years (108). One important reason may be the improper use of intrauterine devices (IUDs) in the early stages of strict policy of the planned parenthood, which led to chronic inflammation and an increase in cancer incidence. Following the WHO’s recommendation to incorporate progesterone into these devices, the mortality rate of endometrial cancer gradually decreased (109). Therefore, it is essential to correctly understand how social factors influence disease trends through population behavior, and to be fully aware that cancer prevention should be prioritized in the implementation of various health-related policies.

## 3 Cancer prevention

**Figure 2:**
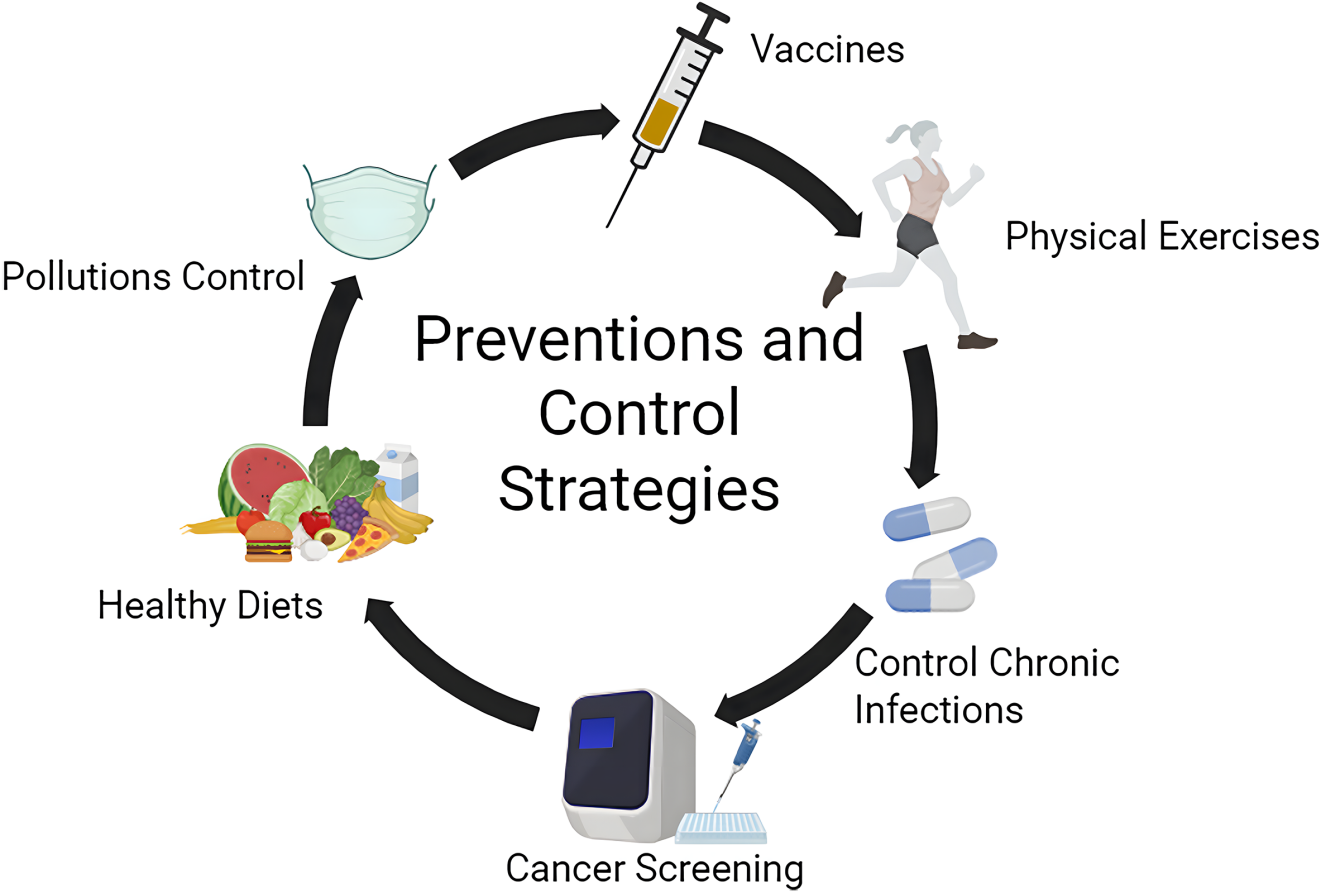
Preventions and control strategies targeting the modifiable risk factors. (Figure was created with https://www.biorender.com, accessed on 5 Febrary, 2025).

About 44.4% of all cancer-related deaths can be attributed to modifiable risk factors, which can be prevented through public health prevention (110) (**Figure 2**). Exposure to these risk factors directly or indirectly induces low-grade systemic inflammation. Beyond direct carcinogenic effects, these risk factors cause persistent tissue and organ damage, leading to necrosis and proliferation, and finally promote the evolution and progression of cancer. The corresponding public health measures mainly include the following aspects:

### 3.1 Physical activity

Chronic systemic inflammation arises from modern lifestyles induced by increasingly specialized division of labor and sedentary job requirements in modern society. This status has become a major cause of cancer, cardiovascular and cerebrovascular diseases, diabetes, and dementia (111–113). Half a century ago, sports medicine posited that intense exercise increased inflammation levels and was harmful to the human body. However, regular physical activity, especially aerobic exercise, has been recently proven to reduce systemic inflammatory responses caused by immune aging dose-dependently (114, 115).

Regular physical activity can lower cancer incidence and mortality by promoting the generation of new immune cells, and increasing several subtypes of T cells, B cells, myeloid cells, and NK cells beneficial to human body (116–119). Regular exercises can also induce muscle-derived cytokines which supports the maintenance of healthy immune effector cell populations (120) and promotes an overall anti-inflammatory milieu (121). Single-cell transcriptomic analysis in animal study revealed that aerobic exercise improves inflammatory pathways in multi-tissue stem cells and their niches in aging animals via anti-inflammatory effects, restores immune cell-mediated intercellular communication, and reshapes their transcriptomic profiles (122). Compared with people who walk 5,000 steps per day, those who walk 10,000 steps per day have a 20% lower incidence of cancer (123). Combining aerobic exercise with muscle-building activities can reduce cancer mortality rates, adjusted for age, gender, and race, by 50% (124).

Therefore, establishing and maintaining exercise habits not only help achieve an ideal physique but also maintain a low-inflammatory state to prevent cancer occurrence and to significantly reduce mortality rates among cancer patients.

### 3.2 Prevention and control of chronic infections

Vaccination is currently the most ideal measure for preventing chronic infections. China implemented a neonatal HBV vaccination program in 1992, which significantly reduced the carriage rate of HBV in the vaccinated population and correspondingly decreased HCC occurrence (125). The currently available HPV vaccine is expected to play a pivotal role in preventing cervical cancer. HCV, an RNA virus with a highly mutable genome, lacks a vaccine, but drugs targeting the viral reverse transcriptase have achieved cure rates of over 99%. Public health prevention targeting the transmission routes of chronic infections is an important measure. HBV is transmitted through mother-to-child transmission, blood transfusion, sharing hygiene instruments, and sexual contact; HCV is mainly transmitted through blood transfusion and injection; HPV is primarily transmitted sexually; *H.pylori* is spread through cross-contamination during food consumption; and liver flukes are transmitted through the consumption of raw infected fish and other aquatic products. Once infected, active treatment is essential to prevent the spread of infection and reduce carcinogenic effects. Therefore, the level of public health services, personal cultivation, and knowledge reserves are key to preventing these cancers.

### 3.3 Pollution control and optimizing living habits

Outdoor or indoor air pollution, soil pollution and water pollution, are not only the causes of chronic inflammation but also directly affect the stability of DNA in human organs through exposure to carcinogenic pollutants. Effective strategies to address environmental health challenges must include public infrastructure investments in water quality, robust air quality standards for pollutants like particulate matter, targeted protection against toxic exposures for vulnerable populations, public education on cancer risk factors (e.g., smoking quitting), and comprehensive environmental self-protection.

To optimize living habits, the following measures are recommended. Avoid consuming unhealthy foods, such as fried, salted, smoked, overprocessed, sugary or high-calories and uncooked foods. Avoid eating foods contaminated with *H. pylori* via saliva or inadequate hygiene. Limit the consumption of fermented and spoiled foods like stinky mandarin fish and fermented tofu. Avoid excessive and long-term alcohol consumption. Establish healthy diet habits which include consumption of fresh vegetables and fruits to supplement vitamins, trace elements, and dietary fiber; regularly consumption of whole grains to maintain normal bowel function; keeping a balance between caloric intake and expenditure through self-discipline to achieve an ideal physique.

We suggest to being surrounded by positive, optimistic, and open-minded individuals to maintain mental and physical well-being and avoiding elevated inflammation levels. These measures can maximize primary cancer prevention and represent the most cost-effective public health cancer prevention strategy.

## 4 Cancer screening

Currently, the majority of cancers are diagnosed in the intermediate or advanced stages, characterized with limited treatment options and poor prognosis. In contrast, early-stage cancers are generally more amenable to curative interventions and demonstrate better responsiveness to therapy. Therefore, urgent need for early cancer screening strategies is underscored. In addition, a substantial proportion of early-stage cancers may never progress to life-threatening stages (126). A critical premise for effective screening lies in understanding which early-stage cancers are likely to advance to late-stage disease and the temporal dynamics of such progression.

Based on progression rates, cancers can be clarified into three categories: rapidly progressive cancers (e.g., PLC, cholangiocarcinoma, gallbladder cancer, pancreatic cancer, and signet ring cell carcinoma), characterized by early onset and aggressive progression; slowly progressive cancers (e.g., CRC, stomach cancer, esophageal cancer, lung cancer, and breast cancer), which often develop through identifiable precancerous lesions over extended periods; and “lazy cancers” (e.g., prostate cancer and thyroid cancer), where tumors may remain clinically insignificant for prolonged periods. Rapidly progressive cancers render population-wide screening impractical, making primary prevention targeting modifiable risk factors remain the cornerstone (127). Slowly progressive cancers have a slower progression, which enables early screening and is suitable for secondary prevention. “Lazy cancers” do not affect life expectancy and can be managed with tertiary prevention (128). Early detection of slowly progressive cancers can be cost-effective. For example, a multi-center randomized controlled trial demonstrated that once-only flexible sigmoidoscopy screening significantly reduced CRC incidence and mortality over a 21-year follow-up (129). CRC has a moderate degree of malignancy, with increasing incidence and mortality rates in China (7), and a higher proportion of early-stage CRC progress to late-stage cancers.

Thyroid, breast, and prostate cancers are frequently associated with overdiagnosis in clinical practice (130, 131). Globally, 75.6% of thyroid cancer cases involve overdiagnosis (132), with China exhibiting an even higher rate of around 80%. The exceptionally rapid increase in ASIR for thyroid cancer may primarily stem from overdiagnosis (133). A retrospective study revealed that merely 38% of thyroid cancer diagnoses were symptom-driven, suggesting substantial overdiagnosis in asymptomatic populations. While adherence to standardized clinical guidelines could mitigate this issue, the ultimate resolution requires development of reliable diagnostic tools (134). Notably, overdiagnosis frequently leads to overtreatment, posing additional clinical risks. Breast cancer demonstrates similar over diagnosis challenges, particularly among women aged over 70 years in the United States, where overdiagnosis rates range from 31% to 54% (135). The advent of mammography screening, despite its technological advancement as compared with traditional clinical breast examination, has paradoxically contributed to increased overdiagnosis. However, therapeutic advancements have substantially reduced breast cancer mortality rates, suggesting that the benefits of modern screening modalities might counterbalance their overdiagnosis potential (136). This complex interplay indicates that conventional diagnostic approaches may retain clinical value in specific contexts. Taking NSCLC as another example, 30–40 years ago, the proportion of squamous cell carcinoma to adenocarcinoma of NSCLC in China was approximately 80%:20%; currently, the proportion is about 10%:90% (137, 138). Some believe that changes in the carcinogenic components of tobacco due to the use of filters are responsible, but this is not the main reason for this reversed ratio. The primary reason might be the shift in diagnostic methods for lung cancer. In the past, X-ray chest films were relied upon, and lung cancers had to be large enough to be detected; recently, low-dose spiral CT is used, allowing detection of tumors just a few millimeters in size, especially peripheral adenocarcinomas common in non-smoking women (139, 140). Although lung cancer CT screening enables early treatment of tumors, nearly 50% of early lung cancers are over-diagnosed (141), creating psychological stress for individuals with non-progressive lung cancer, which in turn may promote cancer-related deaths due to stress.

## 5 Tertiary prevention of cancer

The treatment and prognostic management of malignant tumors represent the final stage of cancer control efforts and are currently the focus of health investment. This primarily involves improving diagnostic and therapeutic levels through surgery, chemotherapy, radiotherapy, immunotherapy, and targeted therapy to achieve the goal of treating cancer. Over a decade ago, epidemiological data on cancer burden in China showed that the trends in the ASIRs and the ASMRs of several important cancers were consistent (3), indicating that clinical treatment had limited protective effects against cancer-related deaths. Due to flaws with follow-up techniques or other reasons, domestic medical institutions often excessively report low mortality rates for cancer patients. For example, domestic medical institutions commonly report 5-year survival rates of 50%-100% for patients with PLC after surgery (142–144). In our analysis of a longitudinal study on PLC prognosis in Yangpu District, Shanghai, from 2002 to 2010, we found that the 5-year survival rate for patients who underwent radical surgery was 32.64%, as compared with 9.01% for those who did not receive radical surgery (145). This is comparable to the outcomes of early-stage PLC surgery in European medical institutions. However, this ratio does not demonstrate radical surgery is more effective than non-radical surgery. At that time, patients who could be hospitalized and receive surgical treatment were more likely to have a higher socioeconomic status such as more medical insurance coverage, better nutrition supply, and regular physical examinations, while the socioeconomically disadvantaged groups likely faced delayed diagnosis and limited treatment. The mortality rate of PLC in rural areas of China is significantly higher than that in urban areas (7), which illustrates this point. Recent clinical studies have shown that the therapeutic efficacy of cancer has been greatly improved through the renewal of chemotherapeutic drugs, precise radiotherapy, blockade of tumor immune suppression by PD-1 and PD-L1 antibodies, immunotherapy based on chimeric antigen receptor (CAR)-T cell therapy, targeted therapy based on blocking tumor angiogenesis, and autologous stem cell transplantation (146, 147). These advancements have shown particularly significant effects on certain malignant tumors, especially hematological cancers such as lymphoma (148). However, since the main characteristic of cancer as a malignant disease is recurrence and metastasis, accurately assessing postoperative recurrence and metastasis of cancer is key to determining clinical treatment plans. Currently, cancer diagnosis and treatment guidelines in China are mainly derived from related foreign guidelines, and their adaptability to Chinese patient needs further evaluation. To clarify the effectiveness of a particular treatment plan, such as 3-year recurrence rates and 5-year survival rates, and side effects, large-scale clinical research cohorts and randomized controlled trials (RCTs) are needed to strictly control various biases, especially loss-to-follow-up bias and information bias (149). RCTs are the gold standard for evaluating the efficacy and side effects of certain therapies. Strengthening the application of epidemiological methods, especially cohort and epidemiological experimental methods, in clinical research will provide the most accurate reflection of the relationship between medical practice and patient survival benefits. This includes the impact of screening, early diagnosis, and various clinical treatments on patient survival. Providing technical support to significantly reduce premature deaths and excess mortality caused by cancer is essential.

The cancer burden in Mainland China is becoming increasingly heavy, with a significant excess of deaths as compared with developed countries (150). This is a serious public health issue now and for the foreseeable future. Identifying the main causes and actively preventing and controlling controllable factors are the keys to curbing this “flood beast”. As with all disease prevention and control efforts, we elucidate the basic laws of cancer development, and emphasize the cost-effectiveness of cancer control. Integrating upstream cancer prevention with downstream clinical treatment is currently the most effective strategy with the best cost-effectiveness ratio for controlling cancer mortality. This is akin to flood control. Neglecting upstream prevention while only focusing on downstream dam construction leads to more frequent and dangerous flooding. Therefore, it is essential to strengthen upstream cancer prevention efforts to significantly reduce and delay the occurrence of cancer.
